# Objective Characterization of Activity, Sleep, and Circadian Rhythm Patterns Using a Wrist-Worn Actigraphy Sensor: Insights Into Posttraumatic Stress Disorder

**DOI:** 10.2196/14306

**Published:** 2020-04-20

**Authors:** Athanasios Tsanas, Elizabeth Woodward, Anke Ehlers

**Affiliations:** 1 Usher Institute University of Edinburgh Edinburgh United Kingdom; 2 Oxford Centre for Industrial and Applied Mathematics Mathematical Institute University of Oxford Oxford United Kingdom; 3 Department of Experimental Psychology Medical Sciences Division University of Oxford Oxford United Kingdom; 4 Oxford Health NHS Foundation Trust Oxford United Kingdom

**Keywords:** actigraphy, sleep, Geneactiv, posttraumatic stress disorder, wearable technology

## Abstract

**Background:**

Wearables have been gaining increasing momentum and have enormous potential to provide insights into daily life behaviors and longitudinal health monitoring. However, to date, there is still a lack of principled algorithmic framework to facilitate the analysis of actigraphy and objectively characterize day-by-day data patterns, particularly in cohorts with sleep problems.

**Objective:**

This study aimed to propose a principled algorithmic framework for the assessment of activity, sleep, and circadian rhythm patterns in people with posttraumatic stress disorder (PTSD), a mental disorder with long-lasting distressing symptoms such as intrusive memories, avoidance behaviors, and sleep disturbance. In clinical practice, these symptoms are typically assessed using retrospective self-reports that are prone to recall bias. The aim of this study was to develop objective measures from patients’ everyday lives, which could potentially considerably enhance the understanding of symptoms, behaviors, and treatment effects.

**Methods:**

Using a wrist-worn sensor, we recorded actigraphy, light, and temperature data over 7 consecutive days from three groups: 42 people diagnosed with PTSD, 43 traumatized controls, and 30 nontraumatized controls. The participants also completed a daily sleep diary over 7 days and the standardized Pittsburgh Sleep Quality Index questionnaire. We developed a novel approach to automatically determine sleep onset and offset, which can also capture awakenings that are crucial for assessing sleep quality. Moreover, we introduced a new intuitive methodology facilitating actigraphy exploration and characterize day-by-day data across 49 activity, sleep, and circadian rhythm patterns.

**Results:**

We demonstrate that the new sleep detection algorithm closely matches the sleep onset and offset against the participants' sleep diaries consistently outperforming an existing open-access widely used approach. Participants with PTSD exhibited considerably more fragmented sleep patterns (as indicated by greater nocturnal activity, including awakenings) and greater intraday variability compared with traumatized and nontraumatized control groups, showing statistically significant (*P*<.05) and strong associations (|*R*|>0.3).

**Conclusions:**

This study lays the foundation for objective assessment of activity, sleep, and circadian rhythm patterns using passively collected data from a wrist-worn sensor, facilitating large community studies to monitor longitudinally healthy and pathological cohorts under free-living conditions. These findings may be useful in clinical PTSD assessment and could inform therapy and monitoring of treatment effects.

## Introduction

### Background

Posttraumatic stress disorder (PTSD) is a mental disorder with lifetime prevalence ranging from 1.9% [1] to 8.8% [2]. However, these figures are considerably exacerbated in conflict, torture, and rape survivors [3,4]. PTSD may develop following exposure to a *traumatic event*, which is defined as *exposure to actual or threatened death, serious injury, or sexual violence* in the Diagnostic and Statistical Manual of Mental Disorders 5th edition (DSM-5) [5]. It is characterized by four symptom categories: (1) re-experiencing symptoms associated with the trauma, which include intrusive memories of the event, (2) avoidance of stimuli associated with the trauma, (3) negative alterations in cognition and mood, and (4) alterations in arousal.

Sleep disturbances have been termed the *hallmark* of PTSD [6] but are still poorly understood. They often have a chronic course and are among the most distressing symptoms [7]; these include difficulties falling and staying asleep [8] and nightmares [9]. Poor sleep has been associated with daytime PTSD symptom exacerbation and severity [10,11], indicating a potentially vicious maintenance cycle between sleep disturbances and PTSD symptoms [11], and highlights the importance of investigating sleep to potentially inform targeted treatment [12]. Crucially, from the perspective of this study, the monitoring environment for symptom assessment is critical: PTSD patients report sleeping better in lab settings than at home [13], where problems are more commonly detected [14,15]. This emphasizes the importance of conducting community studies under free-living conditions instead of lab-based assessments. Moreover, given that PTSD is a chronic condition, it would be desirable to develop low-cost monitoring systems that could provide insight into the patient’s daily behaviors longitudinally; ideally, such systems would require minimum input from participants.

Longitudinal monitoring of mental disorders is typically achieved using the patient-reported outcome measures (PROMs), where participants are prompted to complete standardized validated questionnaires capturing generic or disease-specific symptoms [16-18]. For example, sleep disturbances are common in mental disorders such as depression and PTSD and cause considerable distress and disability. Sleep diaries are recommended as the gold standard for subjective prospective sleep monitoring [19]. Sleep diaries are typically completed over 1 week, asking participants to report the following (among others): time to bed, how long it took to fall asleep, length and number of awakenings, wake-up time, and sleep quality [19]. However, sleep diaries are inherently limited by relying on an individual’s ability to estimate their own sleep times [20]; similar to PROMs, they may be subject to recall bias [21].

Therefore, although self-assessments have merit, they are by nature subjective and may not be easily comparable across individuals [22]. On the other hand, objective monitoring of daily behaviors may offer an additional dimension to understanding pathologies (here, PTSD) using passively collected data. In addition to overcoming the aforementioned problems, sensor data can be streamlined and facilitate direct comparison across participants. There have been considerable developments in the wearable sensor market over the last 5 years; in particular, wrist-worn sensors are widely available and are becoming increasingly affordable. Some have functionalities such as displaying time and communicating with smartphones to convey notifications; in short, these devices are becoming attractive beyond a research exercise, and they are generally embraced by the public. Although many companies have a business model where they employ proprietary algorithms and only offer access to processed data (eg steps, number of stairs, and sleep characteristics such as sleep onset and sleep offset), there are some devices that offer access to the raw *actigraphy* (3D acceleration) data, often complemented with additional modalities such as light, temperature, and heart rate. Overall, the raw signals from the wrist-worn sensor provide a rich source of information and researchers can develop algorithms tailored to the application at hand (often fusing different modalities). For example, actigraphy can be used to objectively quantify physical activity, which can be used as a biomarker for well-being [23]. Although it is currently not possible to obtain accurate estimates of sleep architecture and sleep stages using actigraphy, it provides a good measure of general sleep characteristics including sleep efficiency, total sleep time, sleep onset latency, and awakenings after sleep onset times [24]. Recent work emphasizes participant adherence and the potential of using wrist-worn accelerometers to collect actigraphy data in large-scale population studies [25]. Actigraphy has been used in people with PTSD [10,26] and has shown consistency with sleep diary reports in a PTSD population [27].

Unfortunately, the research literature discussing algorithmic tools to process the raw actigraphy data is limited and fragmented; there is no agreed data-processing protocol and frequently, actigraphy analysis is completed manually relying on visual inspection. This is because studies often have very different focus in terms of applications, for example, to assess general physical activity [25,28] or sleep timings [29], and it is still early days for trying to standardize the commonly extracted features to characterize actigraphy data. Most studies focus on developing and validating actigraphy algorithms using only healthy control cohorts. However, it has been shown across a range of applications that developed algorithms on healthy controls may fail to generalize sufficiently well in cohorts with pathologies [30-32].

### Objectives

This study offers a principled framework summarizing many of the known algorithms and introducing new algorithmic approaches to extract potentially useful information from the actigraphy data. Although the application of this study is PTSD, the developed methodology is generic and, in principle, applicable to settings where actigraphy data are available. To the best of our knowledge, this is the first study to investigate a large set of activity, sleep, and circadian rhythm patterns using a relatively large number of both healthy controls and a mental disorders group where participants commonly exhibit severely fragmented sleep and sleep disturbances.

The aims of this study were to (1) develop an automated approach to accurately determine sleep onset, sleep offset, and awakenings from passively collected data using a wrist-worn sensor targeting a cohort in which sleep is often disturbed, validating findings against participants’ sleep diaries; (2) explore differences in these sleep characteristics under free-living conditions between PTSD participants, traumatized controls, and nontraumatized controls; and (3) methodologically contribute toward a large set of activity, sleep, and circadian rhythm patterns to objectively characterize daily behaviors and develop a simple to use package to facilitate actigraphy analysis in MATLAB (The MathWorks Inc).

## Methods

### Study Cohort

The study cohort comprised 115 participants: 30 nontraumatized controls, 43 trauma-exposed without PTSD, and 42 with PTSD. Inclusion criteria for all groups were as follows: aged between 18 and 65 years, could read and write in English, had no history of or current bipolar or psychosis, no current substance or alcohol dependence, and if they were taking psychotropic medication they had been on a stable dose for at least two months. Additional inclusion criteria for the PTSD group were a traumatic event experience as defined by Criterion A of the DSM-5 [5] and a current diagnosis of PTSD. Inclusion criteria for the control group were no experience of a Criterion A traumatic event and no current mental health problems. Inclusion criteria for the trauma control group were a Criterion A traumatic event experience but not meeting the criteria for a current diagnosis of PTSD. Table 1 summarizes the demographic characteristics of the study cohort. The groups were age and gender matched, and among trauma survivors (n=85), there was no difference in trauma type (interpersonal vs noninterpersonal) between PTSD and trauma controls.

The study received National Health Service (NHS) ethical approval from the South-Central Oxford C Research Ethics Committee (Ref 14/SC/0198). Recruitment began in June 2014 and ended in April 2016. Control and traumatized control participants were recruited via advertisements within the University of Oxford on the departmental website and online community forums in Oxford. The Clinical-Administered PTSD Scale [33], the gold standard for the assessment of PTSD symptom severity, was conducted by EW or JS to establish a PTSD diagnosis according to DSM-5 [5] criteria. All participants completed a sleep questionnaire at trial onset and, over the course of a week, wore a wrist-worn device and kept a detailed sleep diary (see details below).

**Table 1:** Demographic information for the study participants (N=115).

Demographics		Nontraumatized controls (n=30)	Trauma exposed controls (n=43)	Posttraumatic stress disorder (n=42)
Age (years), mean (SD)		31.17 (10.38)	34.02 (14.01)	32.51 (9.93)
Females, n (%)		23 (76.7)	31 (72.1)	26 (61.9)
**Trauma type^a^**, n (%)****				
	Interpersonal	N/A^b^	14 (32.6)	20 (47.6)
	Not interpersonal	N/A	29 (64.4)	22 (52.4)
Time since trauma (years)^c^, mean (SD)		N/A	10.12 (10.18)	8.23 (9.71)

^a^Trauma type and characteristics are only for trauma survivors (n=85).

^b^N/A: not applicable.

^c^Time since trauma was calculated as the time (years) from trauma to study participation date.

### Sleep Questionnaire: Pittsburgh Sleep Quality Index

Sleep quality was assessed using the standardized Pittsburgh Sleep Quality Index (PSQI) questionnaire [34]. The PSQI is a self-report assessment comprising 19 items that are mapped onto seven components (each scored in the range 0 to 3): (1) subjective sleep quality, (2) sleep latency, (3) sleep duration, (4) habitual sleep efficiency, (5) sleep disturbances, (6) use of sleeping medication, and (7) daytime dysfunction. The sum of the 7 subscale component scores generates the total score, known as *total PSQI*, which has a range of 0 to 21; scoring above 5 is used as a standard threshold to indicate poor sleep [34]. The study participants self-assessed the occurrence of sleep disturbances over the previous month on a scale from 0 (not during the last month) to 3 (3 or more times a week) for each item. PSQI was collected once in this study and serves as an overall indication of sleep quality.

### Sleep Diary

A sleep diary was used to prospectively monitor participants’ sleep over 7 days. Participants completed the diary in the morning answering questions about their sleep the previous night. Specifically, they recorded time they got into bed, time they started trying to sleep, sleep onset duration and wake-up time, and number of awakenings (including approximate times), known as wake after sleep onset (WASO). We clarify that by sleep onset, we used the time that participants recorded as falling asleep (ie, time they switched the lights out and started trying to sleep plus reported sleep onset latency). The sleep diary followed recommendations for sleep research [20] and for the prospective self-monitoring of sleep [19].

### Wrist-Worn Device

Participants wore a triaxial accelerometer (Geneactiv, ActivInsights Ltd) on their nondominant wrist for 7 days, coinciding with the sleep diary recording. A recent study endorsed recording at least six nights of standard actigraphy measurements for a reliable measure of self-reported sleep [35]. The device recorded 3D acceleration data at a sampling rate of 100 Hz with a dynamic range of ±8 g (g is a gravity unit, 1 g=9.81 m/second²) and 12-bit resolution. In addition to triaxial acceleration, light (Lux) and wrist temperature (°C) were recorded. We obtained actigraphy data from 113 participants (the missing data were from 2 PTSD participants).

### Methods to Process Data

The following sections describe the methodology used to process the wrist-worn data and statistical tools to compare cohorts.

#### Calibration and Data Preprocessing

Accelerometers convert mechanical force into electrical signals; in practice, they require calibration to ensure that their outputs are directly comparable. Electrical signals are considered a linear function of the acceleration, involving an offset and a gain factor. In some of the older devices, researchers developed algorithms to compute the calibration values [36]; for the Geneactiv used in this study, the manufacturer provides the offset and the gain factor for the 3 axes for each device. We applied the standard calibration procedure using the manufacturer provided offset and gain factors.

Subsequently, the data were resampled at 10 Hz to simplify further processing. Before extracting data patterns, we automatically detected nonwear times, so that these segments could be excluded from the analysis. The nonwear times were determined following a similar process to Zhou et al [37], marking nonwear periods lasting at least 15 min. The argument is that shorter nonwear periods are unlikely to have a marked effect on the results and attempting to determine very short nonwear times would increase the number of segments mistakenly assigned to be nonwear times.

#### Data Visualization and Exploration

We produced three main plot types to facilitate visualization and exploration of the data: (1) *Data summary plot*, simultaneously presenting all the raw signal modalities collected (3D acceleration, temperature, and light); (2) *Actogram*, where stacked plots depict activity over 24 or 48 consecutive hours (in the latter case, there is a 24-hour overlap between successive plots) on successive days; (3) *Colored actogram*, which is like an actogram, but here we express the average level of activity on a 10-min window using a color scale to facilitate direct comparison of activity across days.

A critical intermediate step before further visualization and processing of the data requires summarizing the raw triaxial data. Previous research suggests that there are different approaches but no unique single best way to summarize the activity [38,39]. Here, we define two simple summary measures to achieve this, the *movement* and the *xyz variation* variables, which are both expressing activity in 1-min time windows for time-efficient processing and visualization. The *movement* variable is defined as the square root of the sum of the squared successive differences of the triaxial acceleration data. This is similar to the standard Euclidean distance using the 3D data, with the additional twist that successive differences of the raw acceleration entries are used for each of the 3 axes. We have found that empirically this led to more visually appealing results as we are interested more in the *changes* of position in the 3D space. The movement variable is used to efficiently summarize the data and is the key variable we use to succinctly present activity. We also summarize the raw actigraphy data by introducing the *xyz variation*, which we defined as the rolling 10-min median of successive 1-min acceleration differences with a 90% overlapping window.

We have overlaid the nonwear duration times and sleep duration time (see the following section for details) with transparent color in the data summary plot and the actogram. The data summary plots were further annotated using the sleep diary data provided by the participants. In all cases, the aim was to identify trends in the data and develop an intuitive understanding of the continuity and stability of the emerging patterns.

#### Automatically Detecting Sleep

Previous research has proposed methods to automatically detect sleep using actigraphy, but these are typically evaluated only on healthy controls. Intuitively, we can consider that sleep can be fundamentally determined using actigraphy data on the basis of *sustained inactivity*. Different algorithms proposed in the literature essentially differ on how inactivity is quantified and the use of empirical thresholds, for example, see [29,40,41]. We remark that the latter two approaches were developed using *counts*, a proprietary device-specific estimate of activity. Although there has been a relatively recent attempt to provide backward compatibility with count-based schemes [42], in principle, it would be better to develop approaches using the raw actigraphy data as suggested by van Hees et al [29]. van Hees et al [29] proposed quantifying angular arm movement and assigning time segments to denote sleep when the angle is lower than 5 degrees for 5 successive minutes or more.

The sleep detection algorithm proposed in this study is somewhat more sophisticated. First, we computed the time segments that are considered *sleep candidates* on the basis of the following empirical rules that must be jointly true:

- The rolling 10-min median movement variable is lower than 0.07.
- The rolling 5-min average xyz variation is lower than 0.1.
- The rolling 5-min average light is lower than 30 Lux.

Subsequently, we used a postprocessing approach where sleep candidate segments of at least 30 min in duration were joined if they differed by up to 30 min (to form a continuous sleep candidate segment). The intervening period was recorded as an *awakening* and used to characterize sleep. Finally, all sleep candidate segments less than 2 hours were removed (the threshold might be shortened if we were interested in capturing accurately shorter sleep during the day, at the risk that sedentary activity might be mislabeled as *sleep*). The proposed sleep detection approach requires the availability of ambient light data (some older actigraphy devices do not collect ambient light), but we have found that including this additional modality overcomes problems with sedentary activity which might otherwise be mislabeled as *sleep*.

#### Evaluation of Sleep Detection Accuracy

Lacking polysomnography (PSG), which is the gold standard method for thorough assessment in sleep medicine to determine sleep and sleep architecture [32], we evaluated the accuracy of the proposed sleep detection algorithm using the participants’ sleep diaries. We aimed to demonstrate its competitiveness against the algorithm by van Hees et al [29] (which had been previously validated against PSG). For the algorithm of van Hees et al [29], we used their implementation in the GGIR package [43]. In all cases, we report the difference between the algorithm’s estimate and the ground truth for the purpose of validation (sleep diary).

#### Characterizing Activity, Sleep, and Circadian Rhythm

The detailed equations for the computation of the extracted patterns are provided in Multimedia Appendix 1. The patterns along with a short description are summarized in Table 2. We remark that the categorization of the features into three groups (*activity*, *sleep*, and *circadian rhythm*) is for reporting convenience, and different categorization approaches or feature membership into these categories are possible. For example, M10, L5, RA, IS, and IV characterize diurnal rhythm behaviors and could be assigned to the circadian rhythm category, as some previous studies have suggested [44,45]. The computation of the features can be achieved using any preprocessing approach that summarizes the raw actigraphy data into a vector. Here, we used the *movement* variable. Alternative approaches using other variables that summarize the 3D actigraphy data would also be possible; we defer further elaboration regarding preprocessing for the Discussion.

**Table 2:** Summary of activity, sleep, and circadian rhythm patterns used in this study.

Category^a^ and pattern		Description
**Activity**		
	M10	Average activity for the 10 most active hours
	M10 time	Start time of 10 most active hours
	L5	Average activity for the 5 least active hours
	L5 time	Start time of 5 least active hours
	RA	Relative amplitude of most and least active hours
	MDA	Mean diurnal activity (rise time to bed time)
	MNA	Mean nocturnal activity
	MA	Mean activity with diurnal and nocturnal components
	Diurnal skewness	Skewness of the probability distribution of diurnal activity
	Percentiles diurnal activity	5, 25, 50, 75, 95 percentiles of diurnal activity
	% nocturnal activity (% NA)	Ratio of nocturnal activity over sum 24-hour activity
	IS1	Interday stability using 1-hour windows
	IS2	Interday stability using 1-hour windows with 30-min overlap
	IV1	Intraday variability (24 hours)
	IV2	Intraday variability (1440 min)
	IV3	Intraday variability (24 hours with 30-min overlap)
	Activity TKEO diurnal	Computing the diurnal activity variability using the Teager-Kaiser Energy Operator (TKEO)
	Activity ratio TKEO	Ratio of diurnal activity variability against overall activity variability evaluated using TKEO
	Activity RMSSD	Computing the diurnal activity variability using the root mean squared successive differences (RMSSD)
	Activity ratio RMSSD	Ratio of diurnal activity variability against overall activity variability evaluated using RMSSD
	CMSE	Composite multiscale entropy, evaluating the complexity of the time series at 5, 30, 60, 120 min
**Sleep**		
	Sleep onset	Time starting sleep
	Sleep offset	Wake up time
	Sleep duration	Duration of main (nocturnal) sleep
	Number wake-up	Number of wake-up periods during sleep
	Wake after sleep onset (WASO) minutes	Minutes awake interrupting sleep
	Sleep entropy	Entropy of the activity during sleep (variability of activity during sleep)
	Percentiles sleep activity	5, 25, 50, 75, 95 percentiles of activity during sleep
	Awakenings total minutes	Total number of minutes awakenings lasted for each automatically detected nocturnal sleep
**Circadian rhythm**		
	Sleep temperature zenith	Maximum temperature during sleep
	Sleep temperature zenith time	Time of maximum temperature during sleep
	Sleep temperature nadir	Minimum temperature during sleep
	Sleep temperature nadir time	Time of minimum temperature during sleep
	Sleep temperature range	Range of temperature during sleep
	Sleep onset phase	Successive differences in sleep onset timing
	Sleep offset phase	Successive differences in sleep offset timing
	Cosinor: MESOR	Cosinor model: average measure of rhythm
	Cosinor: Amplitude	Cosinor model: amplitude of fitted sinusoid
	Cosinor: Phase	Cosinor model: phase of fitted sinusoid

^a^For algorithmic details, see Multimedia Appendix 1. Overall, we have 49 extracted patterns (counting the percentiles and the CMSE entries separately). We remark that the categorization of the patterns into the three groups (*activity*, *sleep*, and *circadian rhythm*) is for reporting convenience.

#### Activity

Accelerometers have been traditionally used to quantify physical activity, with an increasing number of wearables and smartphone apps capitalizing on the acceleration signals. There is a growing body of research literature on characteristics that can be extracted from raw actigraphy to quantify activity. Further details regarding the algorithmic expressions for the activity patterns extracted in this study are found in Multimedia Appendix 1.

#### Sleep

We developed a new approach to detect sleep, which was presented in the section *Automatically Detecting Sleep*. Subsequently, we extracted patterns to quantify nocturnal sleep, including awakenings, disturbances, and periods of excessive activity during sleep, which are of clinical interest in PTSD. The algorithmic details for the sleep patterns are presented in Multimedia Appendix 1.

#### Circadian Rhythm

Given the reported chronic course of sleep problems and general long-term effects in daily life, we can use the actigraphy, light, and temperature data to extract *circadian rhythm* disturbances, that is, processes with 24-hour oscillations. The term *circadian rhythm*, strictly speaking, refers to endogenous, entrainable processes; chronobiologists prefer the use of the more general term *diurnal rhythm* to describe self-sustained, repeated processes with 24-hour oscillations when their endogenous nature cannot be confirmed. Given that we are extracting both temperature and activity patterns, we will use the former expression as an umbrella term for simplicity.

A standard approach is to measure core temperature and identify minimum and maximum values over consecutive days [46]. Here, we only have access to wrist temperature, but we wanted to test whether there were any intrinsic differences observed in PTSD. Moreover, we computed differences in terms of the activity phase and sleep phases over consecutive days. Algorithmic details of the extracted circadian rhythm patterns are presented in Multimedia Appendix 1.

### Statistical Analysis

This section describes the statistical tools used to visualize and compare the findings across the three cohorts.

#### Density Plots and Statistical Hypothesis Testing

We computed the densities of the extracted patterns using histograms for discrete random variables and using kernel density estimation with Gaussian kernels for continuous random variables. For discrete random variables, we used the chi-square test to determine whether the distributions are statistically significantly different at *P*=.05 level. For continuous random variables, we used the 2-sample Kolmogorov-Smirnov goodness-of-fit statistical hypothesis test to determine whether the distributions are statistically significantly different, assessing statistical significance at the *P*=.05 level. The null hypothesis was that the samples are drawn from the same underlying distribution. In all cases, we aimed to assess whether there were statistically significant pairwise differences between the three groups.

#### Statistical Association Between Patterns and Groups

We computed pairwise statistical associations between the summarized patterns and groups to quantify differences. Specifically, we computed point-biserial correlation coefficients and used the standard empirical rule of thumb approach that correlations with a magnitude larger than 0.3 are *statistically strong* [47,48].

### Data Accessibility

Requests for access to the data can be made to EW, but the data cannot be placed into a publicly accessible repository.

### Source Code Availability

The MATLAB source code for the computation of the actigraphy patterns will be made available on the first author’s website [49].

## Results

### Self-Reported Sleep: Sleep Questionnaire and Sleep Diary

**Figure 1:**
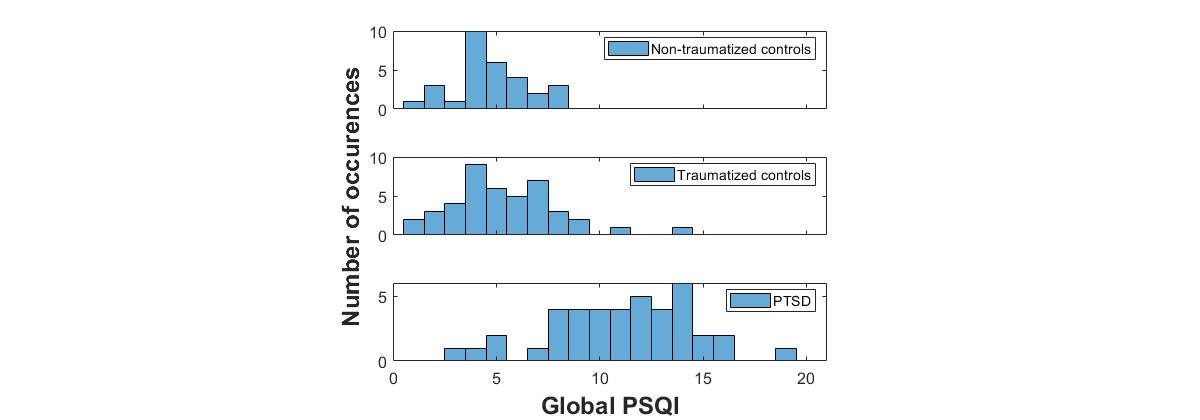
Histogram of the total Pittsburgh Sleep Quality Index scores for the three cohorts in the study. PSQI: Pittsburgh Sleep Quality Index; PTSD: posttraumatic stress disorder.

People with PTSD reported more severe subjective sleep disturbances (higher total PSQI scores) compared with both trauma controls and controls (Figure 1). The PSQI was statistically significantly different between PTSD participants and nontraumatized controls (*P*<.001), and also between PTSD participants and traumatized controls (*P*<.001). There was no statistically significant difference in terms of PSQI between traumatized controls and nontraumatized controls (*P*=.73). In the PTSD group, 91% (38/42) of the participants had clinically marked sleep problems at baseline (total PSQI>5).

### Detection of Sleep Onset and Offset With Automatic Algorithms Compared With Sleep Diary Data

Visual inspection of the sleep diaries and comparison with the actigraphy data revealed that a few participants recorded sleep times that differed considerably compared with the standard sedentary activity typically observed in actigraphy data: this may highlight inherent limitations of sleep diaries [20]. Therefore, we wanted to exclude participants where the difference was markedly clear that it seemed possible that the sleep diary entry was not reliable. This was assessed by visual inspection of the actigraphy data and superimposing self-reported sleep entries. In total, 17 out of the 113 participants were excluded from this assessment: 7 due to lack of valid sleep diary entries for at least three days, 1 due to fewer than 3 days of valid actigraphy data, and 9 due to consistent major disagreement between actigraphy and self-reported sleep entries (also see Multimedia Appendix 1). We clarify that by sleep onset, we used the time that participants recorded as falling asleep (not the time they reported as going to bed or starting trying to sleep, in most cases, there was a couple of minutes difference known as *sleep onset latency*).

**Figure 2:**
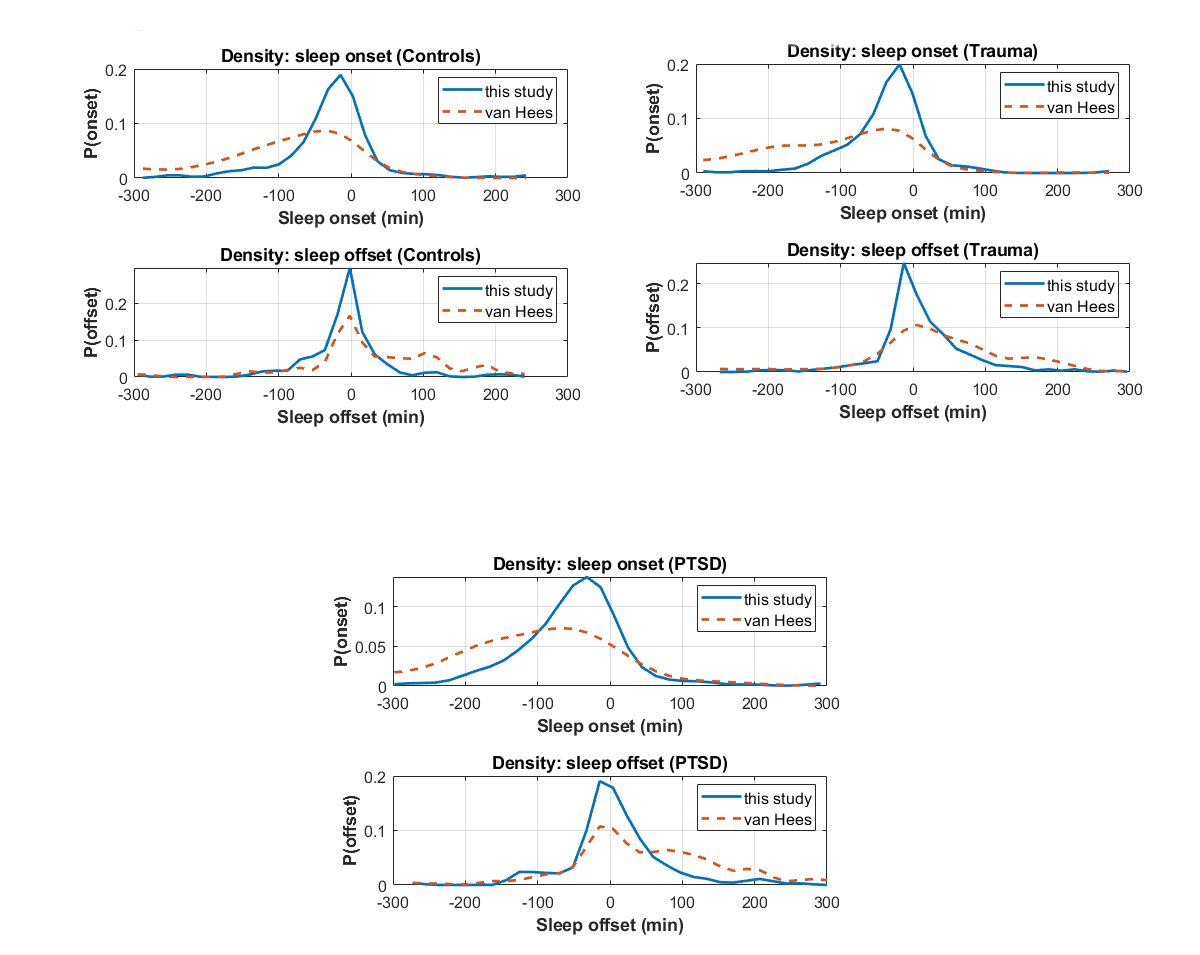
Error density plots comparing side-by-side the estimated sleep onset and sleep offset of the proposed sleep detection algorithm and the sleep detection algorithm by van Hees et al against the sleep diaries, which are used as ground truth. These findings are summarized in Table 3. PTSD: posttraumatic stress disorder.

The agreement of the automatic algorithms against the participants’ sleep diaries in terms of sleep onset and sleep offset is summarized in Table 3. For convenience, we summarize the statistical distributions of the errors (differences in minutes between the algorithmic estimates and sleep diaries) in the form median (IQR). Furthermore, Figure 2 presents the error density plots comparing side-by-side the estimated sleep onset and sleep offset of the new proposed sleep detection algorithm and the sleep detection algorithm by van Hees et al [29], against the sleep diaries that are used as ground truth. We remark that the proposed sleep detection algorithm generalizes sufficiently well for both the nontraumatized controls and the PTSD participants. Overall, the new sleep detection algorithm appears to be very accurate and highly competitive against van Hees’s algorithm, which has been used in some previous studies. As expected, the results are, in general, more accurate for the nontraumatized control group; the PTSD group appears considerably more challenging, likely due to the nature of the disorder affecting sleep, resulting in more irregular patterns, which may complicate sleep detection. Both algorithms can detect sleep offset (wake-up) more accurately than sleep onset. This indicates that people exhibit greater activity in the morning compared with before bedtime, which intuitively verifies what we would have expected.

**Figure 3:**
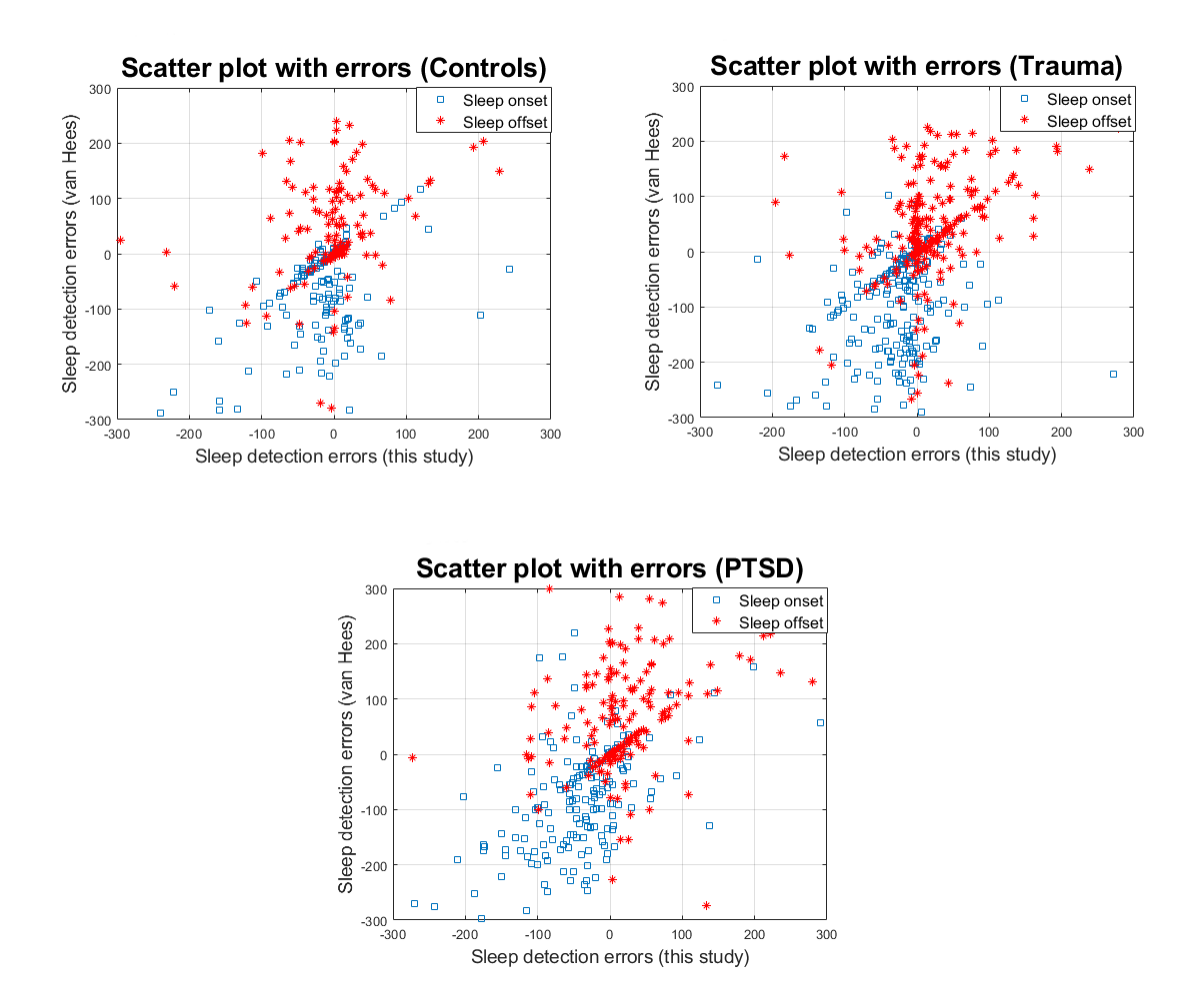
Scatter plots depicting the errors (in minutes) in terms of sleep detection onset and offset across the 3 cohorts for the proposed sleep algorithm against the algorithm proposed by van Hees et al. PTSD: posttraumatic stress disorder.

**Figure 4:**
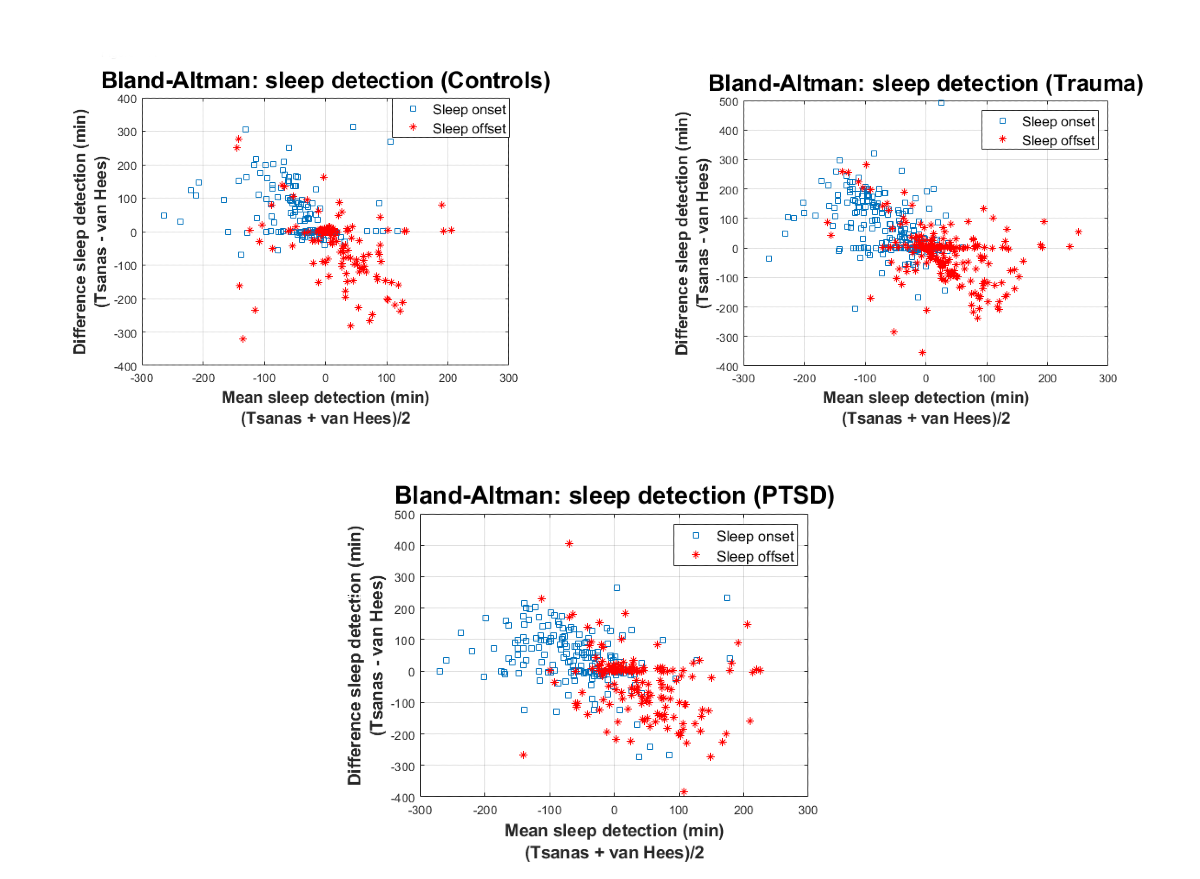
Bland-Altman plots to assess the agreement between the new proposed sleep detection algorithm and the sleep detection algorithm proposed by van Hees et al.

To facilitate direct comparison of the two competing sleep detection algorithms across all samples, we also provide scatter plot results in Figure 3. The algorithm proposed in this study is compared with the algorithm suggested by van Hees et al [29], which had been developed for the programming language R (R Core Team, R Foundation for Statistical Computing). We used standard default settings in the GGIR package developed by van Hees. For both the sleep detection algorithm by van Hees and the sleep detection algorithm in this study, the sleep diary data were used only to compare findings. Subsequently, we also present Bland-Altman plots to assess the agreement between the new proposed sleep detection algorithm and the sleep detection algorithm by van Hees [29] (see Figure 4).

**Figure 5:**
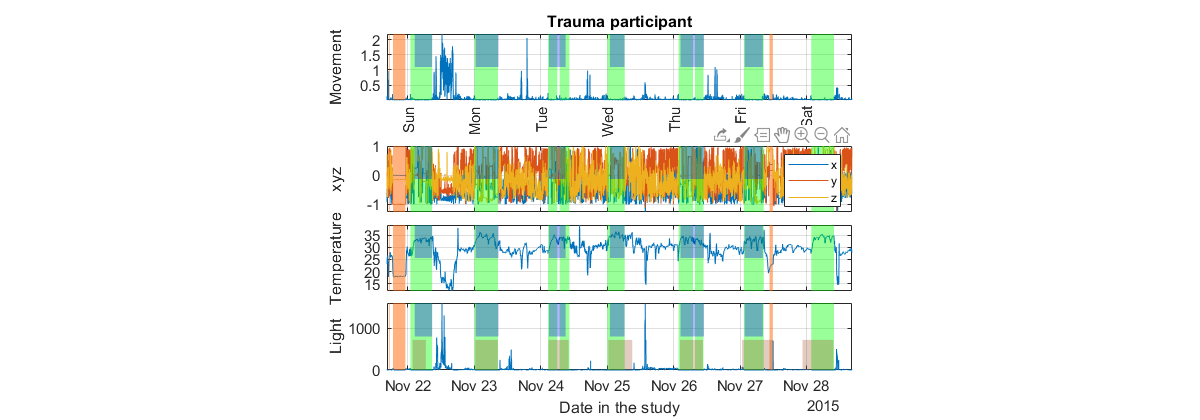
Illustrative indicative example comparing the new sleep detection algorithm with the algorithm proposed by van Hees et al and contrasting findings against the participants’ sleep diary entries (focus on the last subplot). Transparent green indicates the detected sleep times using the proposed algorithm, transparent blue (from midway to top of the plot) indicates the ground truth from the sleep diary, and transparent sienna (from bottom to midway in the last plot) indicates the detected sleep by the algorithm of van Hees et al for comparison. Transparent light brown indicates nonwear times.

Finally, we provide a visual illustration in Figure 5, comparing the algorithm developed by van Hees et al [29] and the algorithm in this study for a randomly selected participant. We observe that the proposed algorithm appears to match very well the participant’s self-reported onset and offset in the sleep diary. We remark that in 2 of the nights during the week this participant was monitored, the algorithm detected awakenings (illustrated with noncontinuous transparent green over the course of the night, indicating broken sleep). The participant had reported awakenings on those 2 nights, although the timings of those awakenings were not recorded. We provide further details about the subplots presented in Figure 5 in the next section.

**Table 3:** Comparison of actigraphy algorithms in accurately detecting sleep: difference in minutes between the algorithms’ estimates and the participants’ self-reports (sleep diaries).

Cohort	van Hees et al [29] sleep detection algorithm^a^		Proposed sleep detection algorithm in this study	
	Sleep onset, median (IQR)	Sleep offset, median (IQR)	Sleep onset, median (IQR)	Sleep offset, median (IQR)
Nontraumatized controls	−56 (112)	22.5 (106)	−12.5 (51)	2 (30.25)
Traumatized controls	−81 (147)	35.5 (95.5)	−18 (50)	10 (46.75)
PTSD^b^ participants	−78 (131.25)	41.5 (122.5)	−34 (78.25)	10 (45.25)

^a^For the algorithm of van Hees et al [29], we used their implementation in the GGIR R package. The results indicate minutes of sleep onset difference and sleep offset difference between the actigraphy algorithm and the ground truth for the purpose of validation (sleep diary). For details on the distributions of the errors, see Figure 2.

^b^PTSD: posttraumatic stress disorder.

### Data Visualization and Objective Outcomes

**Figure 6:**
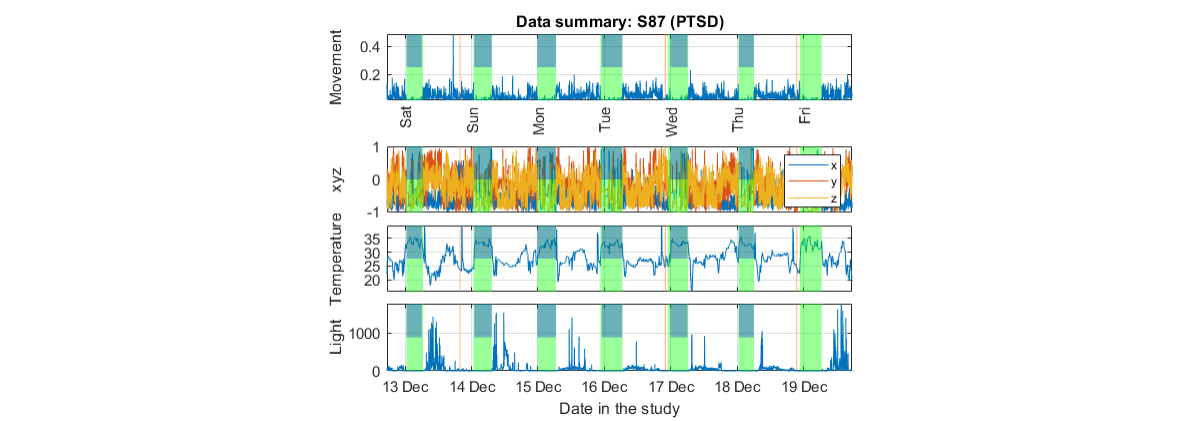
Indicative summary of the collected data for one of the posttraumatic stress disorder participants in the study: 3D acceleration (x, y, z axes), temperature, and light. The first row, movement, is a summary metric of the triaxial acceleration (see text for details). The vertical transparent light green color indicates the automatically assessed sleep times; the transparent light brown color indicates nonwear times. The top midway transparent blue indicates sleep diary entries (which can be used as proxy ground truth). PTSD: posttraumatic stress disorder.

**Figure 7:**
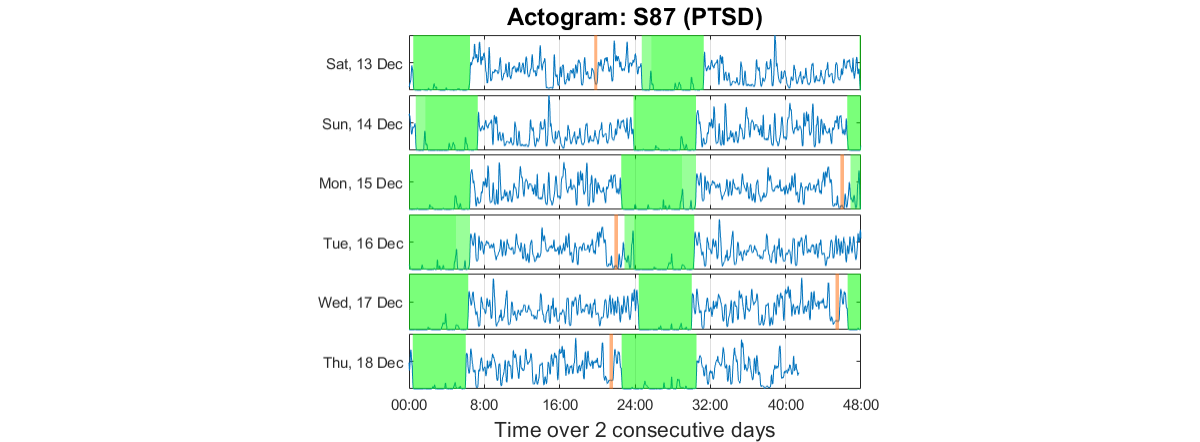
Indicative actogram for one of the posttraumatic stress disorder participants in the study (same participant as in Figure 6). The data on the second half (24:00 to 48:00 hours) of each horizontal plot are repeated as the first (00:00 to 24:00 hours) data on each subsequent horizontal plot; the aim was to have a continuity beyond midnight for the participant. The vertical transparent light green color indicates the automatically assessed sleep times; the transparent light brown color indicates nonwear times. PTSD: posttraumatic stress disorder.

**Figure 8:**
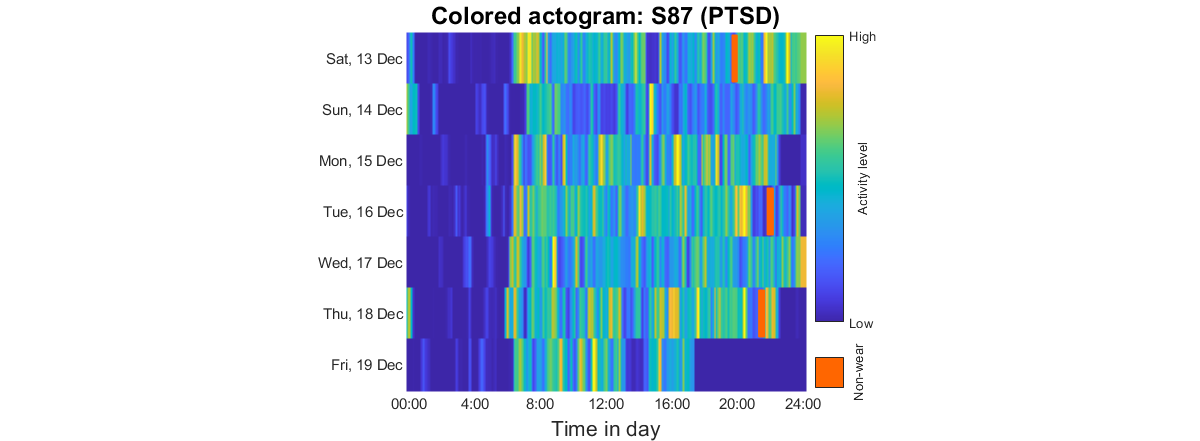
Indicative colored actogram for one of the PTSD participants in the study (same participant as in Figures 6 and 7) to represent activity over 10-min windows. The vertical transparent brown color indicates automatically assessed nonwear times. PTSD: posttraumatic stress disorder.

We present indicative plots for a PTSD participant to illustrate how data can be presented to visualize patterns. Figure 6 shows the data summary plot presenting the raw data, Figure 7 presents the actogram, and Figure 8 illustrates the activity using a color scale. To protect participant anonymity, we have changed the dates in these illustrations.

There are several important insights to be gained by visualizing the raw data in Figure 5: (1) during sleep, the movement is considerably reduced compared with the rest of the day and there are a few large noted changes across 2 axes usually (indicating an occasional, relatively long arm movement during sleep), (2) during sleep the temperature is elevated compared with the rest of the day, (3) we can observe the continuity of activity and sedentary periods during the day, we observe light exposure, which is known to have a strong influence on circadian rhythms (in addition, light can be used to support monitoring wake-ups during the night, eg, to detect toilet visits). The automatically assessed sleep times coincide almost perfectly with the participant’s self-reported sleep diary across all 6 days in Figure 4 (this participant did not provide an entry for the 7th day in the diary). Further examples and detailed results on the comparison of the sleep detection algorithm against sleep diaries and the sleep detection algorithm of van Hees et al [29] are provided in Multimedia Appendix 1.

The actogram in Figure 7 is useful for visualizing the long-term regularity and potential shift changes in activity. Using the sleep annotation (here automatically deduced), we determined the regularity of sleep timings. We also observed movements during sleep, which can be used to quantify sleep quality.

The colored actogram in Figure 8 complements the standard actogram: visualizing the active times of subsequent days once again provides insight into pattern regularity, which can be useful for extracting circadian rhythm patterns. The extracted patterns in the study were motivated in part by visualizing multiple plots of the form presented in Figures 6-8 from the three cohorts.

### Group Differences

Ultimately, each of the extracted patterns characterizes each day, that is, for each participant, we typically have a 49×6 (extracted 49 patterns for 6 days). We summarize these patterns in terms of the mean (SD) values for each pattern to characterize each participant. Table 4 summarizes the statistical distributions of the summarized patterns for the three cohorts, along with pairwise comparisons between cohorts. The direction of the effects can be inferred from the signs of the correlation coefficients: negative signs in the correlations indicate that the summarized pattern generally exhibits lower scores for the first group in the comparison. Overall, we observed that the PTSD cohort exhibited some statistically strong (|*R*|>0.3) differences compared with the nontraumatized control group. For example, the sleep entropy appears to be a good differentiator between PTSD and the nontraumatized control group. Descriptive statistics of the patterns shown in Table 4 for each of the three groups are presented in Multimedia Appendix 1. The direction of the effect can be inferred from the sign of the correlation coefficient.

In Multimedia Appendix 1, we present additional plots to convey a visual impression on indicative differences between a PTSD participant, a traumatized control participant, and a nontraumatized control participant.

**Table 4:** Indicative pairwise statistical comparisons and correlations of the summarized patterns (features) across the three cohorts.

Pattern	Statistical comparisons (*P* values)			Correlations (point biserial correlation coefficient)		
	Control vs trauma	Control vs PTSD^a^	Trauma vs PTSD	Control vs trauma	Control vs PTSD	Trauma vs PTSD
IV2^b^	.06	*.005* ^c^	.06	0.22	*0.33*	0.21
Sleep entropy	*.002*	*.005*	.88	−*0.34*	−*0.32*	0.016
Awakenings total minutes	*.25*	*.01*	.16	0.14	*0.30*	0.16
Number wake ups	.33	*.03*	.06	0.12	0.27	0.21
WASO^d^ total minutes	.41	*.03*	.05	0.10	0.26	0.21

^a^PTSD: posttraumatic stress disorder.

^b^IV2: intraday variability (1440 min).

^c^Statistically significant associations (at the *P*=.05 level) are italicized. We present five indicative summarized patterns that exhibit the largest correlation magnitudes for the binary comparisons between groups. The negative signs in the correlations indicate that the summarized pattern generally exhibits lower scores for the first group in the comparison. Correlations with a magnitude over 0.3 are considered statistically strong. Further details are presented in Multimedia Appendices 1 and 2 (Multimedia Appendix 2 presents all the investigated variables).

^d^WASO: wake after sleep onset.

## Discussion

### Principal Findings

Sensor-based at-home monitoring is rapidly emerging with the proliferation of wearables. Wearables provide a convenient platform for chronic condition management, facilitating detailed longitudinal assessment across diverse metrics, including activity, sleep, and circadian rhythm patterns. This study demonstrated the potential of using raw triaxial acceleration, light, and temperature data to gain insight into weekly patterns of activity and sleep in the participants’ everyday life. We illustrated compact approaches to visualize annotated data modalities and proposed a novel algorithmic approach to estimate sleep onset and offset (including awakenings) that appears to be very accurate against sleep diaries.

We emphasize that the extent of our claim regarding the accuracy of the new sleep detection algorithm is that it replicates reasonably accurately the sleep diaries, which is a proxy for the true sleep onset and sleep offset. Any automated sleep detection algorithm validated on sleep diaries implicitly relies on having accurate labels to report performance and would ideally need to be benchmarked against PSG. We decided to exclude 9 participants from further analysis due to consistent major disagreement between actigraphy and self-reported sleep diary entries following visual inspection of the data summary plots (see Multimedia Appendix 1).

Importantly, some of the actigraphy algorithms were sensitive in showing distinguishable patterns for participants with PTSD compared with nontraumatized controls, which were in line with the self-reported sleep disturbances. These results are summarized in Table 4 and Multimedia Appendix 1. For example, sleep-based features (awakenings, WASO minutes) may provide a useful objective indicator of the degree of restlessness experienced by PTSD patients during sleep. Similarly, the intraday variability (IV2) differentiates well PTSD from nontraumatized controls, possibly indicating bursts of activity in PTSD throughout the day, whereas nontraumatized controls exhibited less activity variability within each day on average. Collectively, these findings are well reflected in the PTSD literature, where PTSD patients are known to have difficulty with sleep, nightmares, and variable sleep [7-10], attributes which are quantified here by the different sleep-related actigraphy metrics. The results provide further important insight into the quality of objective sleep in PTSD. The objective sleep differences are consistent with actigraphy studies that have found differences (such as increased wake after sleep) between people with PTSD and healthy controls [8,50] and between trauma survivors with and without PTSD [51]. However, objective sleep duration did not differ between groups, consistent with some [27], but not all previous actigraphy studies [50]. Therefore, this study adds to the tentative understanding of the nature of sleep disturbances in PTSD by means of the quantified disturbances in the reported patterns and highlights possible targets for potential intervention to further improve comorbid problems during therapy.

We remark that the results reported in Table 4 have not been corrected for multiple comparisons (eg, some researchers use the Bonferroni correction). These corrections are used to reduce type I errors (rejecting the null hypothesis when it is true) but have the important side effect of introducing type II errors (accepting the null hypothesis when it is false). This has urged researchers to suggest that effect sizes, correlation coefficients, and other metrics should be used to support research findings [51]. Here, we report the point biserial correlations for the extracted patterns, some of which appear to denote statistically strong associations (|*R*|>0.3).

The empirical rules and thresholds for the sleep detection algorithm were originally developed by the first author using his own Geneactiv data (n=1, collected for over 4 years) to correctly match onset, offset, and awakenings. From an algorithmic developer’s perspective, using one’s own data has the distinct advantage of cross-referencing activities, knowing the exact underlying ground truth across multiple days (including naps, awakenings, days of bad sleep, etc) and observing the recorded actigraphy signals. The thresholds of the algorithm were adjusted using a small subset (n<10) of the dataset described in this study, but we purposefully did not formally explore in detail optimizing thresholds to correctly match sleep diary entries to avoid overfitting. Ultimately, the sleep detection algorithm proposed in this study will need to be further validated in new cohorts to demonstrate how well it generalizes. The proposed sleep detection algorithm has functionality to detect awakenings, as illustrated in Figure 5. However, we do not have the self-reported onset and offset timings of awakenings; therefore, unfortunately, this is something we could not properly test and validate. Further work is required to demonstrate the potential of the proposed sleep detection algorithm in correctly detecting awakenings and comparing findings against self-reports or preferably PSG. Typically, algorithms developed to process actigraphy signals rely on hard thresholds (including the algorithm by van Hees et al [29]). It is possible that the thresholds chosen for the sleep detection algorithm proposed here might need to be refined in a different cohort (or with a different sensor recording actigraphy signals).

We believe that the colored actogram is a novel, convenient representation to understand longitudinal patterns of behavior. It serves as an intuitive summary of activity levels, potentially highlighting breaks in patterns. In principle, it could also be used as a composite for multiple participants, for example, to visually compare cohorts and identify whether at an aggregate level, there are some consistent patterns of behavior.

### Comparison With Prior Work

The topic of automatically detecting sleep using wrist-worn devices has recently attracted attention [29]. However, the algorithms developed by van Hees et al [29] have not been validated in a group that exhibits considerable sleep problems [29], which is of particular importance in clinical assessment of mental disorders [12]. The new algorithmic approach proposed here to detect sleep has been developed with the aim of being sensitive to cohorts exhibiting perturbed sleep like PTSD and for extracting sleep patterns. In Multimedia Appendix 1, we provided indicative examples of this new approach, and in Table 3 and Figure 2, we demonstrate its competitiveness compared with the algorithm developed by van Hees et al [29]. Nevertheless, we remark that the sleep diaries used to assess sleep onset and wake-up times in this study are inherently limited and subject to participant self-report bias [20]. Therefore, although the current results are extremely promising, more rigorous validation is required to establish the validity of the new sleep detection algorithm against PSG and ideally in a larger sample, including diverse sleep pathologies. PSG provides the objective ground truth for sleep onset, sleep offset, and awakenings: the actigraphy-based patterns extracted computed by GGIR or any algorithmic package cannot be considered as PSG validated. Hence, the focus of using PSG is primarily to assess and compare the accuracy of competing sleep detection algorithms (ideally tested on both healthy controls and people with different sleep disorders). The subsequent computation of sleep-based patterns is heavily dependent on accurate sleep estimates.

High-end equipment has been previously used to provide objective assessment of activity and sleep in mental disorders assessment, including PTSD [52]. PSG is the gold standard for sleep assessment and remains the only accurate approach to gain insight into the actual sleep architecture [32]. However, PSG relies on the use of expensive specialized equipment, and is logistically costly as assessment requires multiple hours of dedicated time by a sleep-certified expert [24]. Standard PSG also requires the participant’s physical presence in the clinic; however, some recent studies have demonstrated the potential of home-based PSG recordings [53]. Similarly, the ActivPAL monitor is considered the gold standard in free-living conditions for step-counting, time spent in sitting/lying, standing, and walking postures [54]. However, it is designed to be worn at the thigh, fixed with a special adhesive pad, and hence is not very practical for long-term use. These difficulties motivate the use of alternative, easy-to-use, more affordable sensors, and wrist-worn watches are prime candidates in that respect. The data in this study were collected using the Geneactiv watch, which has attracted considerable interest across different research groups worldwide [36,42]. A similar popular wrist-worn device that was used, for example, in the UK Biobank study [25], Axivity AX3, has been shown to provide equivalent signal vector magnitude output with Geneactiv [55]; hence, the developed algorithms should, in principle, be directly applicable in studies where that device was used, crucially in the UK Biobank. Similar devices that provide access to the raw triaxial accelerometer data might require some cross-device calibration; otherwise, the developed framework should generalize well. Some older actigraphy devices used *counts* instead of raw acceleration signals and hence tools used to be device specific; although a relatively recent study provides for backward compatibility [42], the trend is moving toward tools that capitalize on the raw actigraphy data.

Summarizing the 3D accelerometry signals in a vector is crucial and is a required preprocessing step in advance of computing the actigraphy patterns (eg, IS, IV, etc). There are many different approaches reported in the research literature but no unique single best way to summarize the activity [38,39]. For example, the Euclidean Norm Minus One [38] is sensitive to calibration errors. Other approaches often rely on short-term windows [39] aiming to smoothen accelerometry fluctuations owing to internal accelerometer noise and hence might not effectively capture transient movements. The proposed approach in this study for the computation of the *movement* as an accelerometry summary, aims to address inherent accelerometer noise fluctuations by effectively operating on successive differences in the raw 3D accelerometer data before computing the Euclidean distance. We tentatively argue that this instantaneous-based approach rather than using local windows might have some advantages in terms of mitigating inherent accelerometer noise. Further work is required to assess whether there is any superior approach toward summarizing 3D accelerometry signals, for example, against a gold standard.

Alternatively, someone might use the subsequent computation of patterns (such as IS, IV, etc) working on each of the different accelerometry summaries (which are used as a preprocessing step) and assess how those patterns might be associated with a clinical outcome. This effectively draws parallels with the feature selection problem in data analytics, lacking ground truth of which are the *true features* and which are predictive of an outcome; researchers apply feature selection algorithms and feed different classifiers. On the basis of the classification performance, they can assess which feature selection algorithms perform best in a given problem [56,57]. We emphasize that for this approach to be valid and generalizable, researchers would need to perform comparisons across different datasets, ideally associating the extracted features with different outcomes. It is also possible that there are different combinations of accelerometry summaries and computation of patterns that work best; future work would be needed to investigate this in more detail.

### Limitations

The primary limitations of this study are (1) the lack of PSG data to validate findings and (2) the study duration of 7 days. With long-term data, more detailed markers of weekly and monthly activity, sleep, and circadian rhythm variability could be developed and further explored. Participants adhered well and wearing the watch was not reported as disconcerting by any participant, which suggests that longer term monitoring may be viable in accordance with a recent study in the UK BioBank [25]. The sample size was sufficiently large for the exploratory aims of this study; nevertheless, larger cohorts might provide better insight into the nature of PTSD. Verification of the developed algorithm for sleep estimation (including awakenings) against PSG would be important in future studies to confirm sleep and wake detection, and further clarify the results. Similarly, we do not have detailed daily self-reported outcome measures (eg, daily mood self-reports as we had used longitudinally in related previous research [18,58,59]) other than the sleep diaries, which could have been associated with actigraphy-extracted patterns and hence further validate the developed algorithms.

The sleep detection algorithm proposed in this study capitalizes on the accelerometry and ambient light modalities; the latter is useful for differentiating sedentary activity and sleep. However, this suggests that the current version of the sleep detection algorithm would not be backwards compatible with devices that only record accelerometry signals. With the sophistication of wearables, additional modalities are becoming available (such as heart rate) and could be harnessed to potentially further improve sleep detection.

More generally, the light sensor modality should be used carefully in the analysis when developing algorithmic tools: lack of detected light does not necessarily indicate that someone is in a dark room. The wrist sensor may hide under a long sleeve, for example, in long-sleeved clothes or pajamas. There may also be abrupt changes in the detected light signal, if the sensor is temporarily visible (or vice-versa blocked).

The study participants wore the standard Geneactiv (Geneactiv Original). The temperature sensor is encased within the waterproof housing of the watch; hence, the recorded temperature depends on how tightly the participants wear the watch: the sensor actually measures a mixture of body and room temperatures. In addition, during the night, temperature changes may (at least partly) indicate that a person has moved their hand above or under the blanket. The wrist temperature recorded has been primarily useful to detect nonwear times and to assess within-person changes, but not for between-person comparison. Researchers wishing to compare recorded temperatures between participants may want to explore a device that provides direct skin temperature recordings such as the Geneactiv Sleep variant [60]. Therefore, the use of the raw temperature measurements in this study should be interpreted very tentatively. For example, the increase in the recorded temperature during sleep seen in Figure 5 is likely a reflection of environmental temperature increase rather than an increase in the wrist temperature of the participant.

### Conclusions

We envisage the developed algorithmic framework laying the foundation for using actigraphy analysis in different settings where raw wrist-worn triaxial accelerometer data are available, aiming to monitor healthy and pathological cohorts longitudinally. We encourage research colleagues to use and expand on the user-friendly MATLAB source code provided in this study to facilitate actigraphy data visualization and analysis. Among mental health conditions, applications to depression are of interest given that low levels of activity and poor sleep are characteristics of the disorder. Future research could investigate extracting additional patterns from the raw signals, potentially complementing it with additional modalities such as heart rate and geolocation, which are embedded in some recent devices. We are currently exploring the potential of using and extending the objective measures provided in this study to monitor longitudinal PTSD behavior, therapy effects, and long-term recovery.

## Appendix

Multimedia Appendix 1 Further details on algorithmic background and additional results.

Multimedia Appendix 2 Statistical comparisons of features across cohorts.

## Abbreviations

DSM-5: Diagnostic and Statistical Manual of Mental Disorders 5th edition
NHS: National Health Service
PROM: patient-reported outcome measure
PSG: polysomnography
PSQI: Pittsburgh Sleep Quality Index
PTSD: posttraumatic stress disorder
WASO: wake after sleep onset
