# Supplementary material for: Objective Characterization of Activity, Sleep, and Circadian Rhythm Patterns Using a Wrist-Worn Actigraphy Sensor: Insights Into Posttraumatic Stress Disorder
Source: JMIR Mhealth Uhealth. 2020 Apr 20;8(4):e14306. doi: 10.2196/14306 (PMC7199134; doi:10.2196/14306)
Supplement: Multimedia Appendix 1 [file mhealth_v8i4e14306_app1.pdf]

Supplementary Material

Objective characterization of activity, sleep, and circadian rhythm patterns using a wrist-worn actigraphy sensor: insights into post-traumatic stress disorder

Athanasios Tsanas[1,2*¥], Elizabeth Woodward[3¥], Anke Ehlers[3,4]

* *Asterisk denotes corresponding author.* (Tel. +44 (0) 131 651 7887)

[1] Usher Institute of Population Health Sciences and Informatics, Medical School, University of Edinburgh, UK

[2] Oxford Centre for Industrial and Applied Mathematics, Mathematical Institute, University of Oxford, UK

[3] Department of Experimental Psychology, Medical Sciences Division, University of Oxford, UK

[4] Oxford Health NHS Foundation Trust, UK

[¥] *A. Tsanas and E. Woodward are joint first authors.*

Emails: (A. Tsanas) Athanasios.Tsanas@ed.ox.ac.uk, tsanasthanasis@gmail.com

(E. Woodward) e.woodward.17@ucl.ac.uk

(A. Ehlers) anke.ehlers@psy.ox.ac.uk

Contact: (A. Tsanas) Usher Institute of Population Health Sciences and Informatics, Medical School, University of Edinburgh, Nine Edinburgh Bioquarter, 9 Little France road, EH16 4UX, Edinburgh, UK

Keywords: actigraphy, acceleration signals, Geneactiv, Post-Traumatic Stress Disorder (PTSD)

Competing interests: We have no competing interests.

1. Characterizing activity, sleep, and circadian rhythm patterns

Following the pre-processing of the actigraphy data as mentioned in the main manuscript, the aim is to extract characteristic patterns. These can be broadly clustered into three categories: activity, sleep, and circadian rhythm patterns.

1.1 Activity patterns

Many studies have focused on extracting Physical Activity (PA) characteristics from actigraphy data, for example see Blume et al. [1]. Traditionally, some of the simplest approaches used are:

- M10, computed as the maximum average activity over 10 consecutive hours in a 24h day. It can be thought of as a measure of diurnal activity during the day. It was computed using minute-wise assessments, as described in Blume et al. [1].
- L5, computed as the least average activity over 5 consecutive hours (this was practically used to determine nocturnal activity without relying on estimates of bed times) in a 24h day. It was computed using minute-wise assessments, as described in Blume et al. [1].
- Relative Amplitude (RA) jointly expresses the information contained in M10 and L5, and has the following form:

$$RA = \frac{(M10 - L5)}{(M10 + L5)} \tag{1}$$

- Mean Diurnal Activity (MDA), mean activity during the 24h when the participant is not sleeping (see the following section for details on how sleep is detected).
- Mean Nocturnal Activity (MNA), mean activity during bed time. We would expect that groups with pathologies which affect sleep may exhibit elevated levels of MNA compared to healthy controls.
- Mean Activity (MA), mean activity across the 24h day considering diurnal and nocturnal components, computed as in Faedda et al. [2]:

$$MA = \frac{\mathbf{x}_{nocturnal} \cdot t_{nocturnal} + \mathbf{x}_{diurnal} \cdot t_{diurnal}}{t_{nocturnal} + t_{diurnal}} \tag{2}$$

where $\mathbf{x}_{nocturnal}$ is the averaged 5 minute epoch activity during sleep, $t_{nocturnal}$ is the sleep duration in minutes, $\mathbf{x}_{diurnal}$ is the averaged 5 minute epoch activity during awake hours, $t_{diurnal}$ is the total time in minutes not spent in sleep.

- Diurnal skewness, is simply the skewness of the probability distribution of activity values during the period from rise time to bed time. It is a standard statistical descriptor which here provides a measure of the asymmetry in the activity. Skewness quantifies the extent to which a probability distribution differs from a normal distribution.
- Percentile diurnal activity, where we compute the 5th, 25th, 50th, 75th and 95th activity percentile from rise time to bed time
- Percent nocturnal activity (%NA), which is the ratio of nocturnal activity over the total sum activity.
- Inter-day stability (IS) expresses the stability of activity across days. It lies in the range 0 to 1, with values close to 1 indicating strong coupling with external *zeitgebers* (sunlight, social interactions). We can reasonably expect that IS would be lower in groups with pathologies compared to healthy controls.

$$IS = \frac{\frac{1}{k} \cdot \sum_{h=1}^{k} (\bar{x}_h - \bar{x})^2}{\frac{1}{1440} \cdot \sum_{i=1}^{1440} (x_i - \bar{x})^2} \tag{3}$$

where $\bar{x}_h$ is the mean activity sampled over k instances, x_i is the activity at the ith minute of the day, and $\bar{x}$ is the overall average activity. We used the classical definition for IS taking averages over 1 hour, and also over 1 hour with 30 minute overlap.

- Intra-day variability (IV) quantifies the fragmentation of the diurnal rhythm and is complementary to IS.

$$IV = \frac{n \cdot \sum_{i=2}^{n} (x_i - x_{i-1})^2}{(n-1) \cdot \sum_{i=1}^{n} (x_i - \bar{x})^2} \tag{4}$$

where n is the number of samples used to analyse the variability for each day that we have recorded data. Here we used $n = 1440$ (using the minute-wise summarized activity), $n = 24$ (using 1-hour summarized activity), and $n = 48$ (using 1-hour summarized activity with a 30 minute window overlap).

- Time dependent Coefficient of Variation (TD-COV), measures the variability across epochs during the period from rise time to bed time, and is computed as:

$$TD-COV = \frac{\frac{1}{(n-1)} \cdot \sum_{i=2}^{n} (x_i - x_{i-1})^2}{\bar{x}} \quad (5)$$

We also borrow standard generic concepts from other fields to introduce new actigraphy measures: the Teager-Kaiser Energy Operator (TKEO), defined in Eq. (6), and the Root Mean Squared Successive Differences (RMSSD), defined in Eq. (7)).

$$Teager-Kaiser\ Energy\ Operator\ \text{(TKEO)} = \frac{1}{n-2} \sum_{i=2}^{n-1} (x_i^2 - x_{i-1} \cdot x_{i+1}) \quad (6)$$

$$Root\ Mean\ Squared\ Successive\ Differences\ \text{(RMSSD)} = \sqrt{\frac{1}{n-1} \left(\sum_{i=1}^{n-1} (x_{i+1} - x_i)^2 \right)} \quad (7)$$

Specifically, we introduce the following new activity measures:

- Activity TKEO, computing the variability of the time series in 5 minute and 30 minute intervals from rise time to bed time. The TKEO has the property of quantifying both amplitude and frequency variation in the time series, and has worked across different applications [3]. Finally, we computed the ratio of diurnal activity over the overall activity:

$$Activity\ \text{ratio TKEO} = \frac{\frac{1}{n_d - 2} \sum_{i=2}^{n_d-1} (x_i^2 - x_{i+1} \cdot x_{i+1})}{\frac{1}{n_d - 2} \sum_{i=2}^{n_d-1} (x_i^2 - x_{i+1} \cdot x_{i+1}) + \frac{1}{n_n - 2} \sum_{j=2}^{n_n-1} (x_j^2 - x_{j+1} \cdot x_{j+1})} \quad (8)$$

where n_d is the number of samples from rise time to bed time, and n_n is the number of samples during sleep.

- Activity RMSSD, computing the variability of the time series in 5 minute and 30 minute intervals from rise time to bed time. RMSSD is another typical simple but useful measure to characterise a time series. Similarly to Eq. (8) we defined also the ratio of diurnal RMSSD over the total RMSSD.

Finally, we introduce the concept of Composite Multiscale Entropy (CMSE) in this domain. It evaluates the complexity of the time series at different time scales. CMSE was proposed by Wu et al. [4] as a more stable alternative compared to the widely used

Multiscale Entropy (MSE), particularly when studying coarse-grained series at a large scale factor (i.e. complexity at distant times compared to the length of the time series). Here, we used it both to evaluate the entire time series (using all days) computing CMSE at 5, 30, 60, 120 minutes, and also to evaluate the complexity of the activity separately for each day at 5, 30, and 60 minutes.

1.2 Sleep patterns

Having extracted the segments corresponding to sleep for each day using the algorithm described in the preceding section, we computed the following:

- Sleep onset, the time detected of (nocturnal) sleep start.
- Sleep offset, the time detected of (nocturnal) sleep end.
- Number of awakenings, number of times detected that nocturnal sleep was interrupted.
- Wake After Sleep Onset (WASO), average time (in minutes) required to go back to sleep after wake, if awakening occurred.
- Sleep duration, the difference between sleep offset and sleep onset minus interim time awake (total duration of awakenings).
- Sleep entropy, the entropy of activity using only the segment marked as sleep time (in general, entropy quantifies the uncertainty (variability) in the data).
- Percentile sleep activity, where we compute the 5th, 25th, 50th, 75th and 95th activity percentile from sleep onset to sleep offset.

1.3 Circadian rhythm patterns

Circadian rhythms impose an approximately 24-hour cycle in the physiological processes of humans, and sleep can be considered to be a consequence of circadian rhythms. Strictly speaking, circadian rhythms are endogenous, entrainable processes; chronobiologists prefer the use of the more general term *diurnal rhythm* to describe self-sustained, repeated processes with 24h oscillations when their endogenous nature cannot be confirmed. We will use the former expression as an umbrella term for simplicity since we use both intrinsic

processes (temperature) and activity to express daily variability measures which we may expect to have a roughly 24h repeating pattern.

Specifically, the following measures were computed:

- Sleep zenith temperature, the maximum temperature during sleep.
- Sleep zenith temperature time, the time that the maximum temperature during sleep occurs.
- Sleep nadir temperature, the minimum temperature during sleep.
- Sleep nadir temperature time, the time that the minimum temperature during sleep occurs.
- Sleep temperature range, the difference between the maximum and minimum temperature values during sleep.
- Sleep onset phase, the difference in sleep onset over two successive nights.
- Sleep offset phase, the difference in sleep offset over two successive nights.

Furthermore, we used the cosinor method to quantify diurnal rhythms by fitting a sine wave to the actigraphy data and computing the Midline Estimating Statistic of Rhythm (MESOR), amplitude and phase. For the algorithmic definitions and further details please refer to Halberg et al. [5] and Refinetti et al. [6].

1.4 Summarizing the extracted patterns

Ultimately, we characterize each Geneactiv recording with 49 patterns (measures) across each of the days data was collected, and hence characterize each participant. The aim is to capitalize on those patterns to get a better understanding into how groups differ and potentially get a tentative insight into PTSD using these objective data to complement self-reported scores. Table 2 in the manuscript provides a succinct summary of the extracted characteristics along with their description (for convenience and easier reference presented here as Table S1).

Table S1. Summary of extracted daily patterns

	Pattern	Description
Activity	M10	Average activity for the 10 most active hours
	M10 time	Start time of 10 most active hours
	L5	Average activity for the 5 least active hours
	L5 time	Start time of 5 least active hours
	RA	Relative amplitude of most and least active hours
	MDA	Mean diurnal activity (rise time to bed time)
	MNA	Mean nocturnal activity
	MA	Mean activity with diurnal and nocturnal components
	Diurnal skewness	Skewness of the probability distribution of diurnal activity
	Percentiles diurnal activity	5,25,50,75,95 percentiles of diurnal activity
	% nocturnal activity (%NA)	Ratio of nocturnal activity over sum 24h activity
	IS1	Inter-day stability using 1 hour windows
	IS2	Inter-day stability using 1 hour windows with 30 minutes overlap
	IV1	Intra-day variability (24 hours)
	IV2	Intra-day variability (1440 minutes)
	IV3	Intra-day variability (24h with 30 minutes overlap)
	Activity TKEO diurnal	Computing the diurnal activity variability using the Teager-Kaiser Energy Operator (TKEO)
	Activity ratio TKEO	Ratio of diurnal activity variability against overall activity variability evaluated using TKEO
	Activity RMSSD	Computing the diurnal activity variability using the Root Mean Squared Successive Differences (RMSSD)
	Activity ratio RMSSD	Ratio of diurnal activity variability against overall activity variability evaluated using RMSSD
	CMSE	Composite Multiscale Entropy, evaluating the complexity of the time series at 5, 30, 60, 120 minutes
Sleep	Sleep onset	Time starting sleep
	Sleep offset	Wake up time
	Sleep duration	Duration of main (nocturnal) sleep
	Number wake-up	Number of wake up periods during sleep
	Wake After Sleep Onset (WASO) minutes	Minutes awake interrupting sleep
	Sleep entropy	Entropy of the activity during sleep
	Percentiles sleep activity	5,25,50,75,95 percentiles of activity during sleep
	Awakenings total minutes	Total number of minutes awakenings lasted for each automatically detected nocturnal sleep

**Circadian rhythm**	Sleep temperature zenith	Maximum temperature during sleep
	Sleep temperature zenith time	Time of maximum temperature during sleep
	Sleep temperature nadir	Minimum temperature during sleep
	Sleep temperature nadir time	Time of minimum temperature during sleep
	Sleep temperature range	Range of temperature during sleep
	Sleep onset phase	Successive differences in sleep onset timing
	Sleep offset phase	Successive differences in sleep offset timing
	Cosinor: MESOR	Cosinor model: average measure of rhythm
	Cosinor: Amplitude	Cosinor model: amplitude of fitted sinusoid
	Cosinor: Phase	Cosinor model: phase of fitted sinusoid

Overall, we have 49 extracted patterns (counting the percentiles and the CMSE entries separately). We remark that the categorization of the patterns into the three groups ('activity', 'sleep', and 'circadian rhythm') is for reporting convenience.

2. Additional results

This section presents additional results to complement findings reported in the main manuscript.

2.1 Sleep detection

Here we present some further illustrations to demonstrate the accuracy of the proposed sleep detection algorithm. First, we present a clear example for a traumatized participant to illustrate how the different raw signals look, focusing on the period during sleep (see Fig. S1). The automatically estimated sleep by the algorithm is presented in transparent green colour, superimposed on the data. The sleep diary entries are presented in the plot with transparent light blue colour mid-way in each plot to facilitate comparison. Another indicative example is presented in Fig. 5 in the manuscript. We observe there is almost perfect agreement between the algorithmic estimate and the sleep diary of the participant regarding the sleep onset and offset times. Studying Fig. S1 confirms intuitive expectations: we observe that during sleep there is relatively little activity. Occasionally, there are large movements across one or two axes, but the frequency of those movements is very low compared to the rest of the day. In general, during sleep the temperature recorded is elevated and exhibits smaller fluctuation. The light level is practically zero. These observations serve to intuitively support the methodologies researchers are using to detect sleep using actigraphy and other modalities. Focusing on the zoomed version of Fig. S1, we

can get an approximate idea for what sleep might look for participants, and hence develop an understanding for assessing possible disagreements between the algorithmic estimates and sleep diaries.

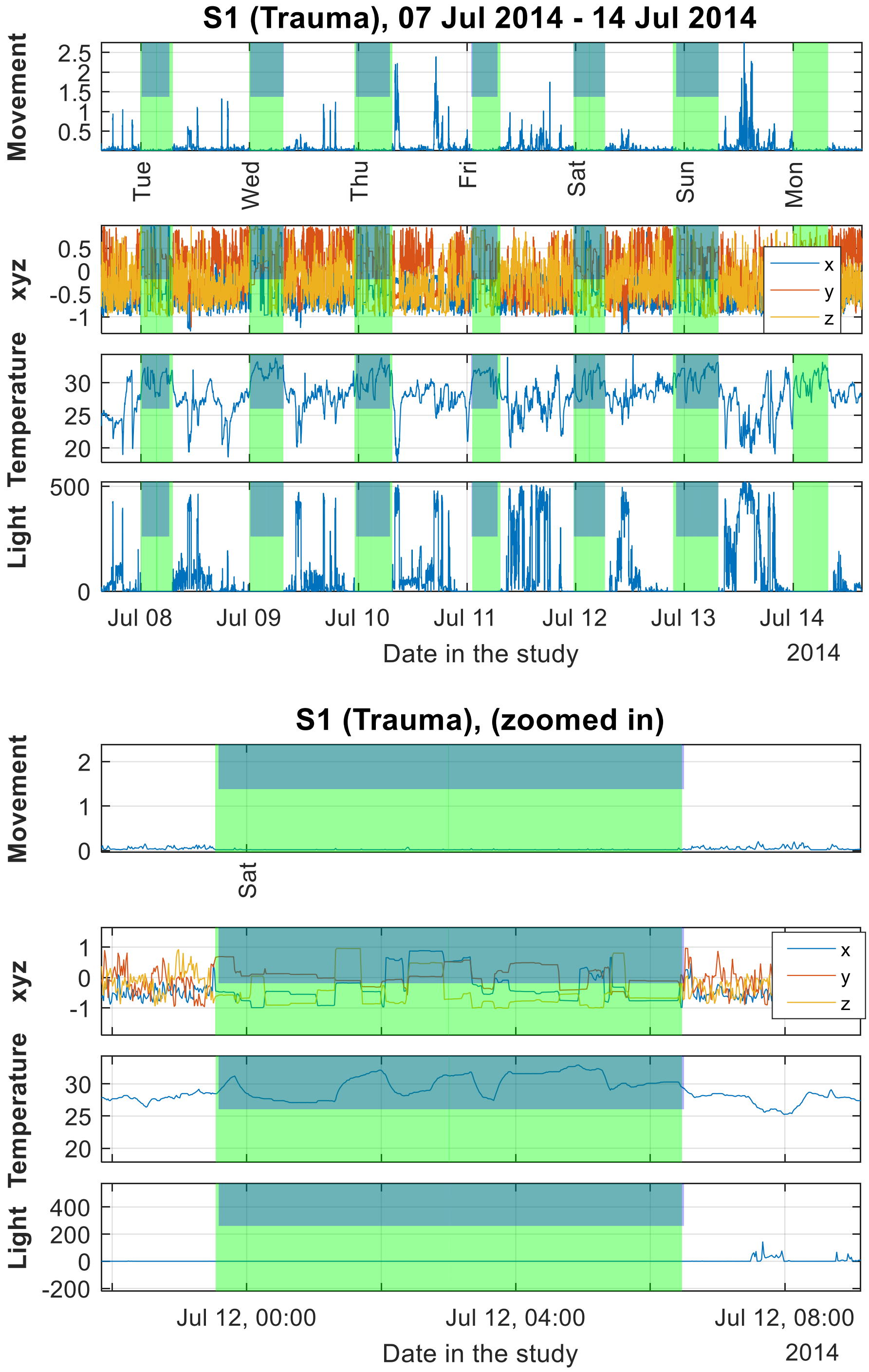

Fig. S1. *Illustration of a clear example of actigraphy signal to detect sleep for a participant. We note that during sleep the activity is considerably reduced, the temperature is in general higher, and there is no light. The vertical transparent light green colour indicates the automatically assessed sleep times; the transparent light brown colour indicates non-wear times. The top mid-way transparent blue indicates sleep diary entries (which can be used as "ground truth"). The first plot presents findings over the entire week, and the second plot is a zoomed in version.*

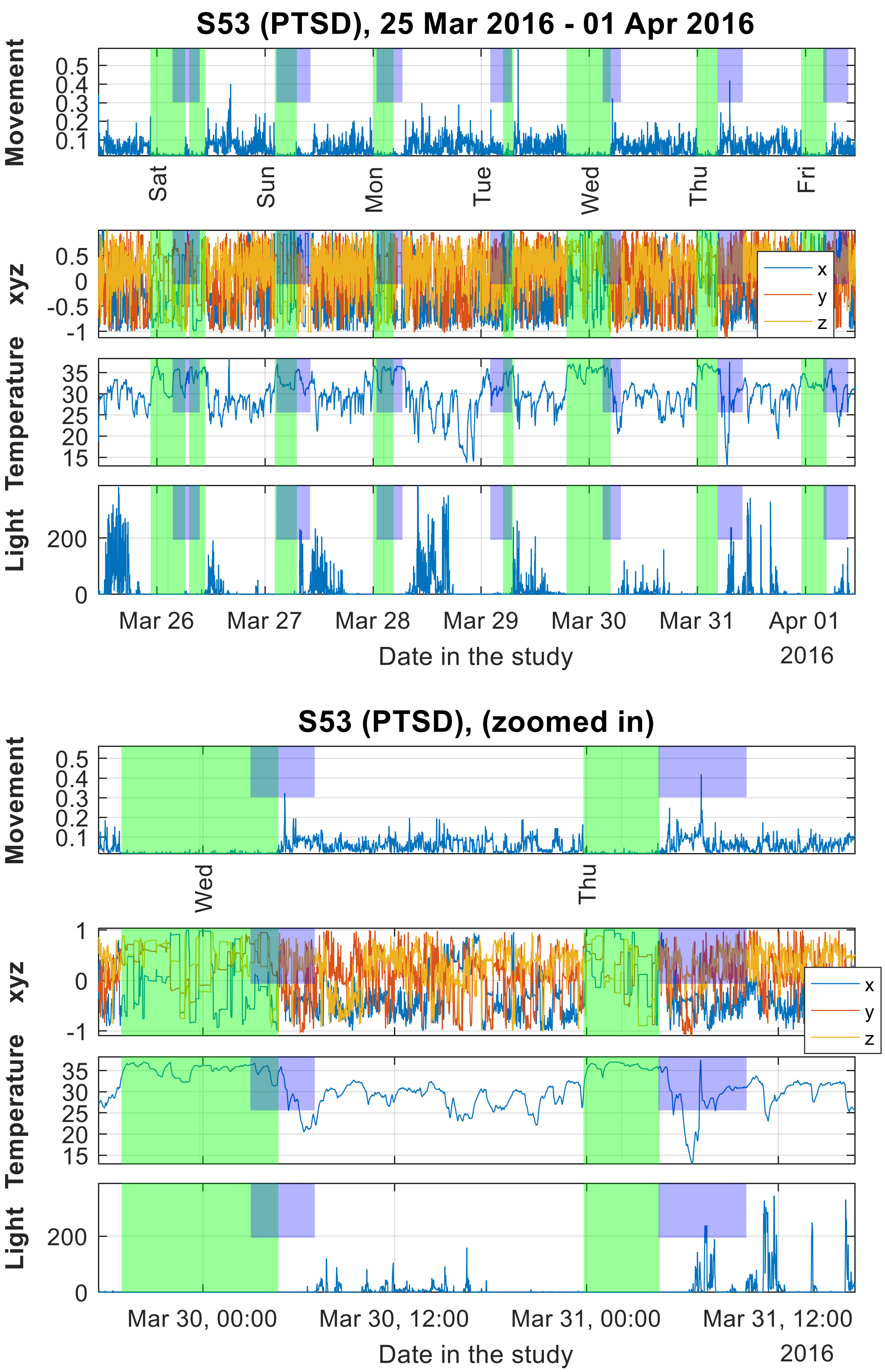

Fig. S2. *Example of major disagreement between the algorithmic estimate of sleep and the sleep diary of a participant. It is clear that the self-reported periods of sleep correspond to periods of physical activity because of the activity patterns and the sharp decrease in temperature. On the other hand, there are periods detected by the algorithm where the actigraphy points quite clearly to sleep.*

Subsequently, we present an example from a participant where it could be argued that sleep appears to be erroneously reported on the basis of the actigraphy data (see Fig. S2). The algorithm has detected periods where the actigraphy visually looks very much like standard sleep (e.g. compare with Fig. S1). In contrast, periods that the participant has marked as sleep appear to be dominated by movement, sharp decreases in temperature, and non-zero light for considerable time, all indicators of standard physical activity. Although this is an extreme example of major disagreement between the algorithmic estimate and the self-reported sleep in the current dataset, it demonstrates a possible problem with self-report and the reliability of certain sleep entries they are when they differ drastically from typical sleep actigraphy patterns.

2.2 Visual comparison of actograms across groups

Figures 5-7 in the manuscript provided indicative plots for a participant. Here, we provide further examples aiming to provide a visual impression of the differences across the three cohorts contrasting the corresponding actograms (see Fig. S3). The actogram of the non-traumatized control provides regular patterns with similar sleep onset and offset times across all days. The traumatized control and the PTSD participant do not have the same consistency as the non-traumatized control, and there is greater variability in circadian patterns. Also, for this PTSD participant the algorithm has detected awakenings over two nights resulting in fragmented sleep.

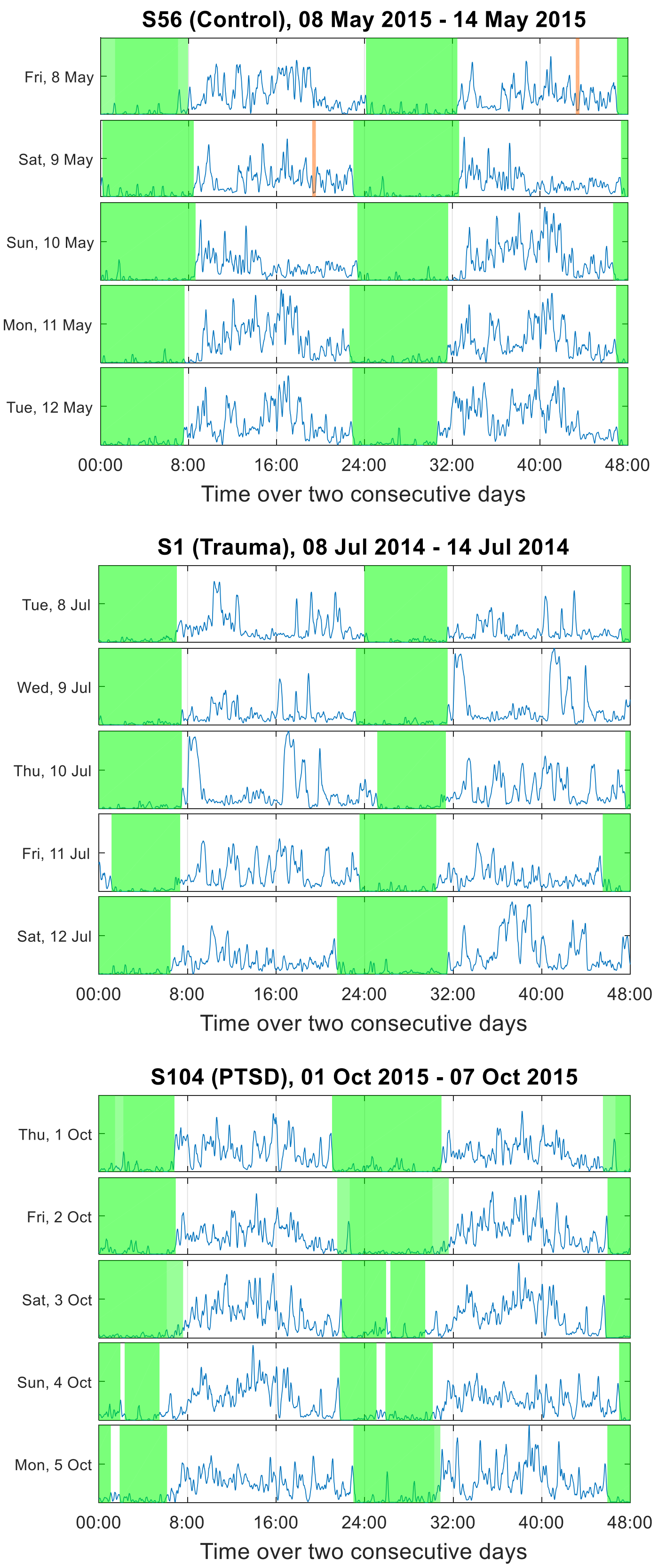

Fig. S3. *Actogram plots of a non-traumatized control, a traumatized control, and a PTSD participant to gain a visual impression of indicative differences across cohorts. Light transparent green indicates automatically detected sleep periods; awakening periods are defined by breaks in continuity of sleep.*

2.3 Descriptive statistics

Table S2 provides an overview of some indicative descriptive statistics of the variables that are reported in Table 4 of the manuscript. We provide a more detailed overview of the descriptive statistics for all variables used in the study in the additional Supplementary Material ‘Statistical comparisons of features across cohorts.xlsx’, which is an Excel file.

Table S2. Descriptive statistics of the five most discriminative patterns

summarized patterns (features) across the three cohorts.

	**Control**	**Trauma**	**PTSD**
IV2	0.428±0.137	0.478±0.110	0.493±0.076
Sleep entropy	5.540±0.218	5.439±0.281	5.376±0.317
Awakenings total minutes	3.000±5.000	5.000±6.833	6.417±7.833
Number wake ups	0.000±0.000	0.000±0.167	0.167±0.167
WASO total minutes	0.000±0.000	0.000±4.500	2.583±8.167

The entries are summarized in the form median ± interquartile range. Statistical comparisons of the results and the reporting of correlation coefficients appear in Table 4 of the manuscript.

References

[1] Blume C., Santhi N., Schabus M.: "nparACT package for R: A free software tool for the non-parametric analysis of actigraphy data", *MethodsX*, Vol. 3, pp. 430-435, 2016 (doi: 10.1016/j.mex.2016.05.006)

[2] Faedda, G.L., Ohashi, K., Hernandez, M., Mcgreenery, C.E., Grant, M.C., Baroni, A., Polcari, A., Teicher, M.H.: "Actigraph measures discriminate pediatric bipolar disorder from attention-deficit/hyperactivity disorder and typically developing controls", *J. Child Psychol. Psychiatry Allied Discip*. Vol. 6, pp. 706–716, 2016 (doi:10.1111/jcpp.1252)

[3] Tsanas, A. Accurate Telemonitoring of Parkinson's Disease Symptom Severity Using Nonlinear Speech Signal Processing and Statistical Machine Learning. Ph.D. thesis, University of Oxford, UK, 2012

[4] Wu, S.-D., Wu, C.-W., Lin, S.-G., Wang, C.-C., Lee, K.-Y.: "Time Series Analysis Using Composite Multiscale Entropy", *Entropy* Vol. 15, pp. 1069–1084, 2013 (doi:10.3390/e15031069)

[5] Halberg F., Tong Y. L., Johnson E. A. The cellular aspects of biorhythms: Symposium on Rhythmic Research Sponsored by the VIIIth International Congress of Anatomy Wiesbaden (8–14 August 1965) 20–48 (Springer, Berlin Heidelberg, 1967)

[6] Refinetti, R., Lissen, G.C., Halberg, F. Procedures for numerical analysis of circadian rhythms. Biol. Rhythm Res. Vol. 38, pp. 275–325, 2013
